# Supplementary material for: qPCR and loop mediated isothermal amplification for rapid detection of Ustilago tritici
Source: PeerJ. 2019 Sep 30;7:e7766. doi: 10.7717/peerj.7766 (PMC6776072; doi:10.7717/peerj.7766)
Supplement: Supplemental Information 1 [file peerj-07-7766-s001.docx]

>JN367307.1

AACTAAACTTGAGCTACCTTTTTCAACACGGTTGCATCGGTCGGCCTGTCAAACAGCGCGACGCAAGGAGAAAATCCTCGCGTCTGCTGGGCGACGGACAGACAATTTTATTGAACACTTTTTGATGATCTAGGATTTGAAGGAGAAAAAGTCATTTTTACGAATGAAATCGACTGGTAATGCGGTCGTCTAATTTTAAAAACAACTTTTGGCAACGGATCTCTTGGTTCTCCCATCGATGAAGAACGCAGCGAATTGCGATAAGTAATGTGAATTGCAGAAGTGAATCATCGAATCTTTGAACGCACCTTGCGCTCCCGGCAGATCTAATCTGGGGAGCATGCCTGTTT

GAGGGCCGCGAATTGTTTCGAACGACAGCTTTTTTCTTTTGGAAAAGGTTGACGGATCGGTATTGAGGGTTTTTGCCATTTATCGTGGCTCCCTTGAAATAGATTAGCGCATCCATTTTATAGGCAAGACGGACGAAAGCTCGATTTTTGCTCTCTCTTCCCTGCCGGGTTTTGATACTATCAGGACTTCGGAGAGGTTGAGATGGGTAGGAGCTCGACGCAACGGCTTGCTGTTTGGAGTGCTTCTGAAACCCGCCCATGCAG
